# Veratridine Can Bind to a Site at the Mouth of the Channel Pore at Human Cardiac Sodium Channel Na_V_1.5

**DOI:** 10.3390/ijms23042225

**Published:** 2022-02-17

**Authors:** Alican Gulsevin, Andrew M. Glazer, Tiffany Shields, Brett M. Kroncke, Dan M. Roden, Jens Meiler

**Affiliations:** 1 Department of Chemistry, Center for Structural Biology, Vanderbilt University, Nashville, TN 37212, USA; tiffany.shields@vanderbilt.edu (T.S.); jens.meiler@vanderbilt.edu (J.M.); 2 Department of Medicine, Division of Clinical Pharmacology, Vanderbilt Center for Arrhythmia Research and Therapeutics, Vanderbilt University Medical Center, Nashville, TN 37232, USA; andrew.m.glazer@vumc.org (A.M.G.); brett.m.kroncke.1@vumc.org (B.M.K.); dan.roden@vumc.org (D.M.R.); 3 Department of Pharmacology, Vanderbilt University Medical Center, Nashville, TN 37232, USA; 4 Department of Biomedical Informatics, Vanderbilt University Medical Center, Nashville, TN 37232, USA; 5 Institute for Drug Discovery, Leipzig University Medical School, 04103 Leipzig, Germany

**Keywords:** voltage-gated sodium channels, cardiac sodium channels, SCN5A, veratridine, toxins, molecular docking, Rosetta, electrophysiology, site-directed mutagenesis

## Abstract

The cardiac sodium ion channel (Na_V_1.5) is a protein with four domains (DI-DIV), each with six transmembrane segments. Its opening and subsequent inactivation results in the brief rapid influx of Na^+^ ions resulting in the depolarization of cardiomyocytes. The neurotoxin veratridine (VTD) inhibits Na_V_1.5 inactivation resulting in longer channel opening times, and potentially fatal action potential prolongation. VTD is predicted to bind at the channel pore, but alternative binding sites have not been ruled out. To determine the binding site of VTD on Na_V_1.5, we perform docking calculations and high-throughput electrophysiology experiments in the present study. The docking calculations identified two distinct binding regions. The first site was in the pore, close to the binding site of Na_V_1.4 and Na_V_1.5 blocking drugs in experimental structures. The second site was at the “mouth” of the pore at the cytosolic side, partly solvent-exposed. Mutations at this site (L409, E417, and I1466) had large effects on VTD binding, while residues deeper in the pore had no effect, consistent with VTD binding at the mouth site. Overall, our results suggest a VTD binding site close to the cytoplasmic mouth of the channel pore. Binding at this alternative site might indicate an allosteric inactivation mechanism for VTD at Na_V_1.5.

## 1. Introduction

### 1.1. Na_V_ Structure and Function

Voltage-gated sodium channels (Na_V_) are ion channels consisting of four transmembrane domains (DI-DIV) containing six segments each (S1–S6) and found in multiple cell types. The S5 and S6 segments assemble to form an ion pore that is responsible for sodium ion conduction through the protein, depending on the voltage changes in the environment as sensed by the S4 segments [1]. Na_V_ play important roles, including setting the membrane potential and initiation of the action potential and the modulation of its duration. As a result of these functions, Na_V_ are targeted for the treatment of multiple diseases, such as epilepsy, pain, cardiac arrythmias, and cancer [2]. Of Na_V_ family proteins, the cardiac sodium channel (Na_V_1.5, encoded by the SCN5A gene) is found in cardiac muscle. Mutations in SCN5A, encoding Na_V_1.5, are the cause of cardiac conditions, such as Brugada syndrome, Type 3 Long QT syndrome, progressive cardiac conduction defect, atrial fibrillation, and dilated cardiomyopathy [3,4].

### 1.2. Na_V_1.5 Activity Is Modulated by Multiple Classes of Drugs

Na_V_ activity can be modulated by several drug types, including anticonvulsants (e.g., phenytoin or carbamazepine), local anesthetics (e.g., procaine or etidocaine), and antiarrhythmic agents (e.g., mexiletine, lidocaine, quinidine, or flecainide). These molecules typically act as channel blockers that reduce ion conductance in voltage-, state-, and frequency-dependent manners [5,6]. Arrhythmic agents are of particular importance because of their therapeutic use. Within this class, the drugs can be further divided based on the Vaughan Williams classification, which divides drugs into 3 classes based on their strength and the ion channel they target [7]. A potential problem with the arrhythmic drugs targeting Na_V_1.5 is the toxicity issues or side effects related to their use [8]. Therefore, alternative molecules that target Na_V_1.5 are desirable to both study Na_V_ mechanism of action and for therapeutic purposes. A mechanistic understanding of Na_V_ activation based on the mode of action of known toxins can help us design better drugs that do not suffer from similar toxicity issues.

### 1.3. Veratridine Is a Plant Alkaloid That Delays Channel Desensitization

**Figure 1:**
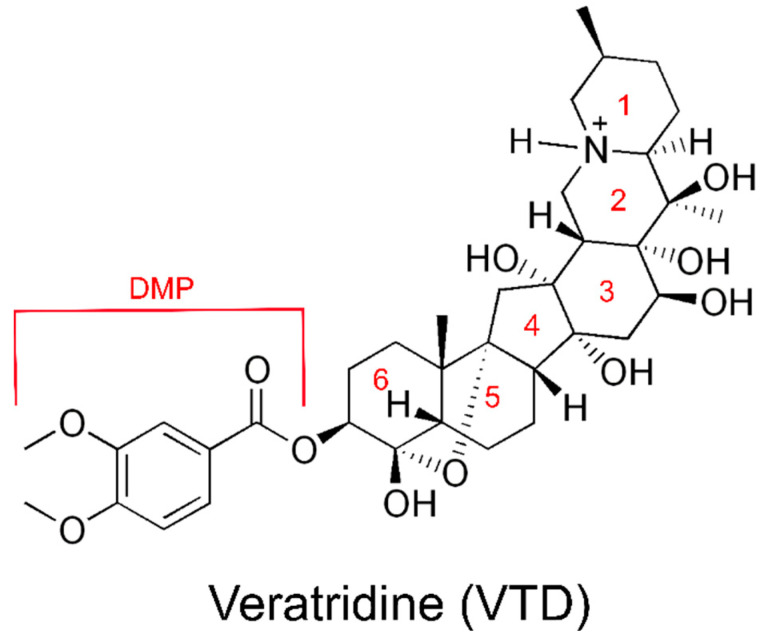
The structure of veratridine (VTD) drawn in its protonated form with the individual rings and the dimethoxyphenyl (DMP) group as denoted in this manuscript labelled in red.

Veratridine (VTD) is an alkaloid from the Veratrum sabadilla plant that binds to Na_V_, including Na_V_1.5 [9]. It consists of six fused rings, in which Rings 4 and 5 are connected through a bridging oxygen to Ring 6. Ring 6 is also attached to a dimethoxyphenyl (DMP) ester group (Figure 1). VTD binds to the open state of Na_V_ [10] and results in slower activation and inactivation of the channels [11]. Due to its prolongation of channel opening times, VTD has been used as scientific tool in studying Na_V_ properties. The use of VTD as a therapeutic agent to treat hypertension was abandoned due to toxicity issues [12]. However, understanding the mechanism of VTD binding to Na_V_1.5 may help us better understand the mechanism behind its delayed activation, and help us develop other drugs that can be used for the treatment of cardiac disorders.

### 1.4. VTD May Bind to Multiple Sites on Na_V_1.5

**Figure 2:**
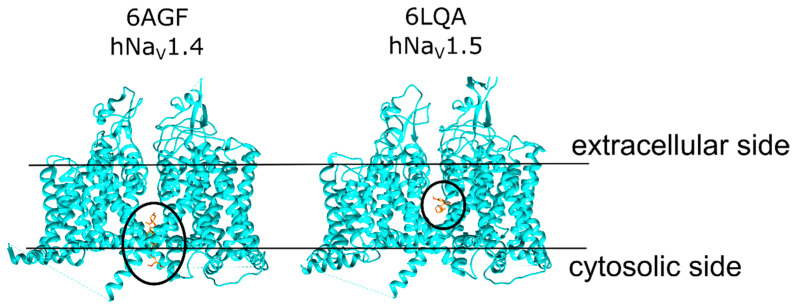
The structures of human Na_V_1.4 (PDB ID: 6AGF) bound to a detergent molecule at the “mouth site” (**left**), and the human Na_V_1.5 (PDB ID: 6LQA) bound to quinidine at the “pore site” (**right**). The ligand molecules are shown in orange for both structures. The approximate region of each binding site is marked with a black circle. The black bar represents the putative position of the membrane with the extracellular and cytosolic sides of the membrane labelled.

VTD is considered to bind to a site between the neuronal Na_V_ domains DI and DIV, close to the intracellular end of the protein [13]. On the other hand, VTD may bind to alternative binding sites, and recent studies suggested a VTD binding site deeper in the pore. A study with Na_V_1.4 proposed a VTD binding site at the pore of this protein based on molecular docking and electrophysiology experiments [14]. In the present study, we tested VTD binding at two sites. The first site is a “mouth” site at the opening of the ion channel on the cytoplasmic face of the protein (Figure 2, left panel). This binding site was proposed based on the Na_V_1.4 cryo-EM structure that had a hydrophobic detergent molecule ((3beta,14beta,17beta,25R)-3-[4-methoxy-3-(methoxymethyl)butoxy]spirost-5-en, ID: 9Z9) bound to the opening of the pore (PDB ID: 6AGF [15]), which may also apply to VTD due to its hydrophobic ring system similar to this detergent molecule. The second site is a “pore” site deeper in the ion channel consistent with the position of blocking drug molecules and that of the putative VTD binding site in Na_V_1.4 calculations (Figure 2, right panel). VTD binding at both sites was first tested with molecular docking calculations and in silico ΔΔG calculations to identify the binding configurations at these sites, followed by electrophysiology experiments on the SyncroPatch high-throughput platform [16] to probe specific residues involved in these interactions.

## 2. Results and Discussion

### 2.1. Veratridine Was Docked to the Mouth Site and the Pore Site

In order to explore whether VTD binding is possible at both the mouth and pore sites, we ran molecular docking calculations with Rosetta, with large grids that are centered at the putative residues of the mouth and pore sites (Supporting Figure S1). Of the 10,000 total poses, the lowest-scoring 1000 poses were clustered into 5 representative poses based on ligand root-mean-square deviation (RMSD) values and ligand interface scores for both the mouth and the pore sites. This approach allowed us to identify the interactions between alternative configurations of VTD and the protein. The resulting poses had a variety of VTD configurations and interactions between this molecule and Na_V_1.5. The pore site scores were lower than the mouth site scores on average, but this difference was less than 1.0 Rosetta Energy Units (REUs) (Table 1). The variation among the mouth site poses were up to 2.5 REU for the lowest- and highest-scoring poses.

### 2.2. Three Pose Clusters Were Identified to Bind Close to the Mouth of the Pore When the Docking Grid Was Centered at the Mouth Site

Pose 1 has Ring 1 facing the direction of the solvent with the DMP ring buried in the pore (Supporting Figure S2A). At the mouth of the pore, VTD interacted with the protein residues S943 and D1471 through hydrogen bonding. Additional hydrophobic interactions were formed with F934, L935, F942, L938, L939, I1470, V1764, and I1771. Y1767 and I1466 formed extensive hydrophobic interactions with the two faces of the DMP ring.

Pose 2 is “flipped” compared to Pose 1, whereby the charged nitrogen is facing the pore and the DMP ring is situated closer to the mouth of the pore (Supporting Figure S2B). All the interactions within 3 Å were hydrophobic, and the charged nitrogen did not form any electrostatic interactions in this pose. L939 interacted with the DMP ring on one side, but there were no interactions formed on the other side. Other hydrophobic interactions of VTD were with L409, L935, L938, I1466, I1470, V1764, and Y1767.

Pose 3 lies on the mouth of the pore without significant interactions with the residues lining the bottom of the previous two poses, such as L409 or I1466 (Supporting Figure S2C). A413, L935, L938, and L939 were close the end of Ring 1, and the DMP ring was exposed to the solvent. A hydrogen bond was formed with D1471 and a VTD -OH group. Other hydrophobic interactions were formed with I1470, I1768, L1772, F1775, and I1771.

### 2.3. Two Pose Clusters Bound Deeper in the Pore with the Charged Nitrogen Facing Opposite Directions inside the Pore

Poses 4 and 5 bound deeper in the pore contrary to the first three poses and had no contacts with the residues lining the mouth of the pore, such as E417 and D1471 (Supporting Figure S2D,E). Pose 4, similar to Pose 2, had the charged nitrogen pointed towards the center of the pore, but it formed no electrostatic interactions with any of the protein residues. The residues T1417, I1756, and S1710 formed the bottom of the VTD binding site, and A413 and L935 formed the top of the binding site. The DMP ring was surrounded by L409, L931, and F934 on the three sides. Additional hydrophobic interactions were formed with L1462, I1466, and F1760. Pose 5 was also deeper in the pore compared to the first three poses, but the charged nitrogen pointed towards the mouth of the pore. The bottom of the binding site was defined by F1760, L1462, F1465, and L1761. There were no residues forming the top boundary of the binding site, but the residues A413, L935, L938, I1470, I1771, and F1775 surrounded Ring 1. More interactions were formed with I1466, Y1767, V1763, L931, L938, and L409.

Overall, the five clusters were inconsistent with each other and suggested different binding sites and different binding orientations with the charged nitrogen placed in different positions. Two of the five poses bound deeper in the pore, two poses bound along the pore, and one pose bound to the mouth of the pore with a tilted angle. Next, we investigated VTD binding to the pore site.

### 2.4. Three VTD Poses at the Pore Site Were Buried Mostly within the Ion Channel and Intercalated the Surrounding α-Helices

Pose 1 runs parallel to the channel pore with the ring group of DMP facing the extracellular side of the membrane (Supporting Figure S3A). This group was surrounded by the residues K1419, E898, E375, A1711, and C373, although none of these residues formed strong interactions with VTD. At the other side of the molecule, the ring that bears the charged nitrogen formed hydrophobic interactions with the residues L1462, F934, and L409. An additional interaction was formed with F1418. Pose 2 lies orthogonal to the channel pore, whereby the DMP group is buried between the residues I1455, L895, F892, V930, L933, and F1459 (Supporting Figure S3B). Ring 1 was buried between the residues I1466, V1764, Y1767, and V1763. Additional hydrophobic interactions were formed with L931, F934, and L1462. The bulk of Pose 5 is inside the ion channel (Supporting Figure S3E). The DMP group was surrounded by V1763, I397, T1709, and it formed a hydrogen bond with Q371. F1760 formed a strong hydrophobic interaction with the core of VTD. Additional interactions were with V1764, L409, I1466, L931, F934, L1462, and F1459.

### 2.5. Two Pore Site Poses Had Tenuous Interactions with the Pore Residues and Interacted Mostly with the α-Helices Lining the Pore

Pose 3 has only Ring 1 inside the channel pore, and the rest of the molecule extended towards the sides of the protein between multiple α-helices that run in the direction between the cytoplasmic and the extracellular sides of the membrane (Supporting Figure S3C). The DMP ring was buried between several residues, including L1342, Y1409, T1753, L1413, W1345, and I1757. A1416, T1417, and S1458 also interacted with the core of the molecule. Different from Pose 3, Pose 4 has the DMP group facing the ion channel, although the interactions are only peripheral (Supporting Figure S3D). This group interacted with F1418, L1462, and F1760. The rest of the VTD molecule was buried between the α-helices akin to Pose 3, where it formed interactions with I1749, L1410, Y1409, T1753, L1342, I1757, W1345, and L1338.

### 2.6. Eleven Mutations Were Selected to Probe the Effect of Na_V_1.5 Mutations on VTD Binding

Based on the poses obtained from different clusters, we chose eleven variants at eight sites. Some of these were designed to remove charged or hydrophobic interactions to probe interactions between these side chains and the VTD poses to eliminate irrelevant docking poses, whereas others were designed to introduce minor changes to the side chain structures to pinpoint the location of specific residue—ligand interactions. It is important to note that considering the large number of residues that interact with VTD in all the different pose clusters, it was feasible to investigate only a fraction of these putative interactions. The residues selected for the experiments were chosen to exploit the similarities and the differences observed between the mouth site and the pore site poses in addition to the configurational differences at each site.

### 2.7. E417 and F942 Were Designed to Selectively Affect Mouth Site Binding

The two E417 mutations (E417A and E417R) were selected to test the effect of mouth site mutations on VTD binding. This residue sits at a solvent-exposed region at the mouth of the ion channel and its mutation would have no effect if the ligand binds to the pore site. E417A was designed to sever hydrogen bonding or electrostatic interactions between VTD and this residue. E417R was designed to block the pore opening due to the larger size of arginine and to cause a clash between the charged nitrogen of VTD if E417 and this nitrogen are in close proximity.

F942 sits close to the opening of the mouth where it forms hydrophobic interactions with a small number of poses. The F942A mutation was selected to rule out these poses, and the F942Y mutation was selected to test whether the additional -OH group could form hydrogen bonds with the -OH groups of VTD in the vicinity, thus improving the binding of VTD.

### 2.8. Four Mutations Were Designed to Diminish Hydrophobic Interactions between Na_V_1.5 and VTD

The L409S and L1462S mutations were selected to check whether these two residues form hydrophobic interactions with the VTD rings that would suffer from the removal of the leucine methyl groups. The I1466T and I1771T mutations also had a similar logic, but this time the focus was on removing the ethyl group only to test the extent of hydrophobic interactions between these residues and VTD. It is important to note that L409 and I1466 are situated in a position that forms the bottom border of the proposed mouth site and the top border of the pore site, therefore these mutations would be expected to be relevant for VTD binding at both sites.

### 2.9. Aromatic Residue Mutations at Two Sites Were Introduced to Alter Hydrogen Bonding, Hydrophobic Interactions, and π-Cation Interactions

F1760 is situated at the pore site below the residues L409 and I1466. This residue interacted with the pore site poses through hydrophobic and π-cation interactions but had no significant interactions with the mouth site poses. The F1760A mutation was chosen to abolish the hydrophobic and π-cation interactions formed between VTD and the pore poses. The F1760Y mutation was chosen to increase the size of the side chain that could interact with the DMP ring of the mouth site poses, and to test whether another aromatic residue is tolerated if VTD binds to the pore site. Likewise, the F1465Y mutation was selected to introduce an additional -OH group that increases the size of the phenylalanine side chain, which was predicted to extend further towards the ion channel and improve VTD binding at the mouth site.

### 2.10. Five Mutations Had a Moderate or Large Effect on VTD Binding

Using automated patch clamping of *SCN5A* mutations expressed in HEK293 cells, we measured the effect of mutations on VTD’s modulation of Na_V_1.5 late current. When added to Na_V_1.5, VTD partially inhibits channel inactivation and increases the Na_V_1.5 late current from minimal (~0.3% of peak current) to large (10–30% of peak current) (Supporting Figure S4A). To quantify the variant effects on VTD binding, we measured the concentrations of VTD necessary to delay the current of Na_V_1.5 by 10% (Supporting Figure S4B). Six out of eleven mutations have little or no effect on VTD binding (<two-fold change in VTD concentration necessary to induce a 10% late current), three mutations have a moderate effect (two- to four-fold change), and two mutations have a large effect in our SynchroPatch experiments (>six-fold change; Table 2).

**Figure 3:**
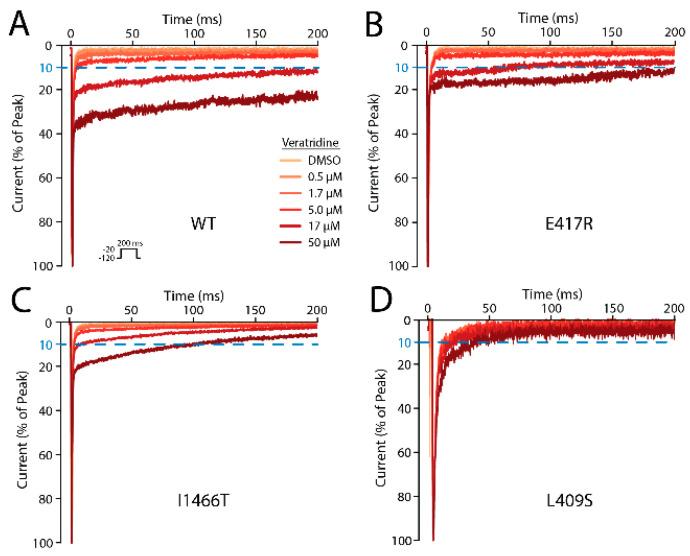
Automated patch clamp measurements of voltage-gated sodium current for representative HEK293 cells expressing wild-type SCN5A (**A**), E417R (**B**), I1466T (**C**), or L409S (**D**). Cells were treated with increasing concentrations of veratridine (inset) and subjected to a repeated voltage pulse (inset). All traces are normalized so that the peak inward current is at 100%. All three variants have a reduced response to veratridine.

The three mutations that have the largest effects on VTD activity are L409S, I1466T, and E417R (Figure 3). The ineffectiveness of the E417A mutation despite the ~four-fold binding decrease caused by E417R suggests that VTD binding to the mouth site can be blocked by the larger arginine side chain, but there are no direct interactions between VTD and E417 that would be severed by the E417A mutation. The lack of a change observed with both F942 mutations rules out mouth site poses close to this residue. The L1462S and I1771T mutations showed that these two residues have no significant role in VTD binding and the introduction of hydrogen bonding partners did not cause the formation of new interactions between these residues and VTD.

The moderate binding diminution caused by F1465Y and F1760Y suggests increasing the residue size close to the ion pore is not well-tolerated. The ineffectiveness of the F1760A mutation rules out a large number of pore site poses that formed hydrophobic and π-cation interactions with this residue. Likewise, the large effect of L409S and I1466T mutations rules out the pore site poses that extend orthogonal to the ion pore with no interactions with these residues. Overall, these results favor VTD binding to the mouth site due to the decrease caused by the E417R mutation and the lack of a binding change due to the F1760A mutation. In addition, the results rule out the mouth site poses that do not contact the residues L409 and I1466. Next, we ran in silico ΔΔG calculations to identify the VTD poses at both binding sites consistent with the measured binding data.

### 2.11. Screening of the 1000 Lowest-Scoring Poses for ΔΔG Changes Identified at the Mouth Site Pose Are More Consistent with the Electrophysiology Experiments

**Figure 4:**
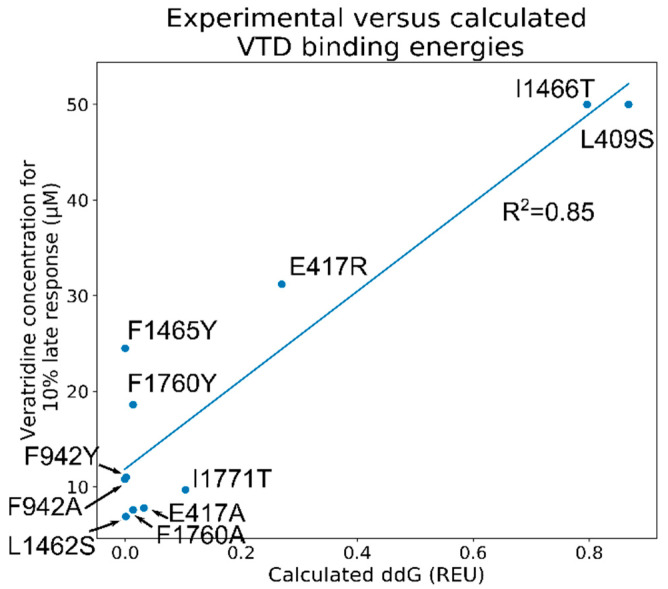
The correlation between the measured VTD concentrations necessary for 10 % delayed late response the ΔΔG values calculated by Rosetta. The experimental concentrations corresponding to the L409S and I1466T mutations were considered to be 50 µM due to the inability to measure their effects at higher concentrations caused by their large responses.

Considering the variability of the binding configurations of the clustered poses at both the pore and mouth sites, we chose to focus on all the top-scoring poses instead of running analyses only on the clustered poses. Specifically, we calculated the ΔΔG values for all 1000 lowest-scoring poses and calculated the Pearson correlation constants between ΔΔG and VTD concentration necessary for a 10% late current. This identified the poses that were most consistent with the experimental data. In order to achieve this goal, we used Rosetta’s *ddGScan* protocol [17,18] without global repacking as a method to screen the ΔΔG values of the selected mutations. The R^2^ values were calculated with Python and these values were used to select the best pose at each site. With this approach, the best pore site poses had an R^2^ value of 0.75, but the calculated scores were close to 0 for most cases and they could not distinguish between the significant and insignificant residues. On the other hand, the mouth site poses have R^2^ values up to 0.85, which also reflect the distribution of the measured binding changes upon mutations (Figure 4). Further, comparison of the three highest-correlation poses show very similar configurations with identical DMP group configurations, consistent with a stable binding pose at this site (Supporting Figure S5A). A closer look at the relation between the calculated and measured binding values showed that the calculated ΔΔG values can distinguish between the high-effect and low-effect mutations, but are unable to distinguish smaller differences as observed for the mutations F1465Y and F1760Y.

### 2.12. The VTD Pose That Showed the Best Correlation Had Interactions Consistent with the Experimental Data

**Figure 5:**
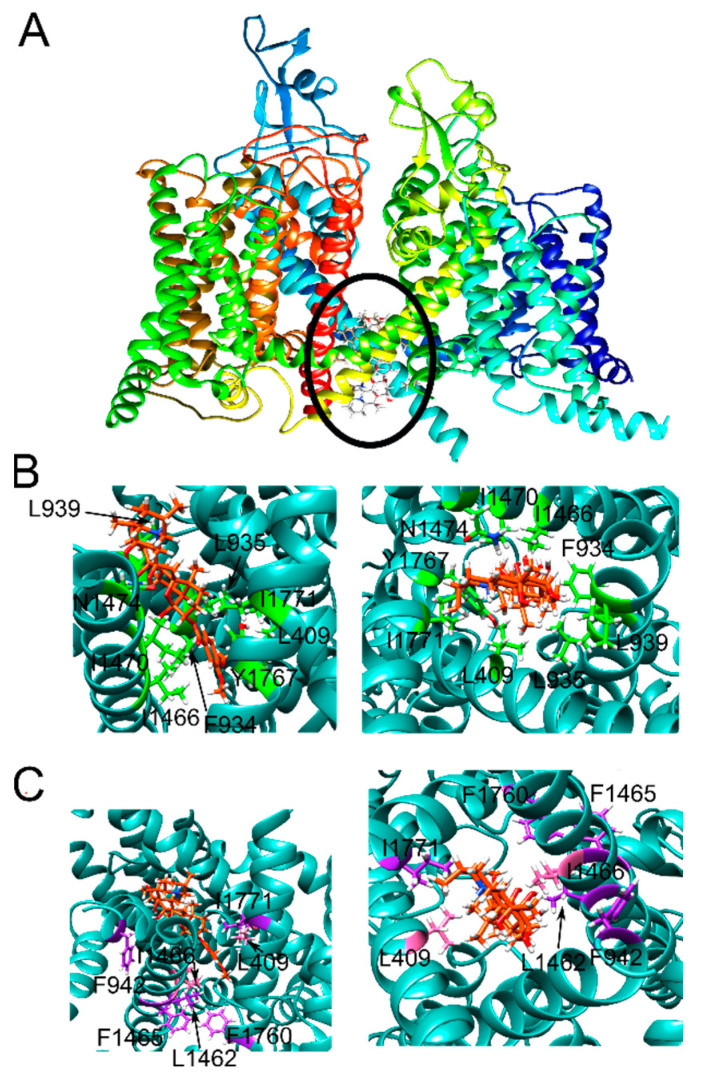
The mouth site pose that had the best correlation factor viewed from different angles. (**A**) A side view of Na_V_1.5 showing the binding configuration of VTD at the mouth site (black circle), whereby the top section corresponds to the extracellular side and the bottom section corresponds to the cytosolic side; (**B**) VTD configuration at the mouth site with the residues reported to form interactions with VTD based on docking calculations labeled; and (**C**) VTD viewed from two different angles, whereby the residues whose mutations showed a significant (pink) and insignificant (purple) change based on the experimental results labeled. VTD is shown in light gray in all panels.

The VTD configuration corresponding to the highest-correlation pose of the mouth site calculations lie along the channel pore with the DMP group pointing towards the center of the channel pore and the charged nitrogen facing the solvent (Figure 5A). The DMP group is surrounded by the residues F934, L935, L409, and I1466, whereby the last two residues are in close contact with the ring of this group on either side (Figure 5B). Y1767 stood closer to the carbonyl group of the ester connecting DMP to the VTD rings. The -CH_3_ group of I1771 interacted with Ring 6 of VTD, although the I1771T mutation had no effect on VTD binding in our experiments. L939 was situated between Rings 4 and 5, and I1470 interacted with Ring 6. Closer to the opening of the pore, N1474 formed a hydrogen bond with an -OH group at the carbon connecting Rings 3 and 4. As predicted, there are no significant interactions with the residues L1462, F1760, F942, and F1465 (Figure 5C). There was no interaction with E417, whose mutation to an arginine resulted in a loss of binding, but the distance between the carboxyl oxygens and the closest VTD atom was ~2.5 Å, which is consistent with diminished activity caused by an arginine mutation at this site. A comparison of the highest-correlation pose with the second and third highest-correlation poses shows similar VTD conformations (Supporting Figure S5B). Overall, this binding pose at the mouth site is consistent with the binding data obtained through electrophysiology experiments and suggests an alternative binding site for VTD at Na_V_1.5.

### 2.13. Delayed Activation and Channel Block by VTD May Work through Two Distinct Mechanisms

VTD activity at Na_V_1.4 was investigated previously through computational and experimental methods, to explore the roots of the agonism and antagonism shown by VTD at this protein [14]. The interactions identified at the binding site by docking were tested by electrophysiology experiments to confirm the involvement of these residues. Two types of VTD effects were investigated: agonism as measured by the activating effect of VTD on Na_V_1.4, and antagonism as measured by the effect of VTD on maximum peak current. The docking site investigated in this study with Na_V_1.4 overlapped parts of the pore site and the mouth site investigated in our study. The residues identified to be important in the Na_V_1.4 study were L1280 (Na_V_1.5 L1462), F1283 (Na_V_1.5 F1465), I1284 (Na_V_1.5 I1466), F1579 (Na_V_1.5 F1760), N1584 (Na_V_1.5 N1765), and F1236 (Na_V_1.5 F1418).

The most drastic diminution of VTD agonism was caused by the L1280K, F1579A, F1236K, and N1584A mutations as measured by the percent of receptors bound to VTD. L1280K, N1584A, and F1579A also caused a large reduction in current peak along with the mutation F1283A. Of the equivalent positions in Na_V_1.5, only the first two were tested, where the L1462S and F1760A mutations had little effect on delayed activation, and F1760Y had a minor effect. The difference in the L1462 results can be explained by the formation of additional hydrogen bonds in the case of L1462S to compensate for the lost hydrophobic interactions of leucine, while the L1462A mutation is incapable of forming hydrogen bonds and the long sidechain of L1462K can easily block VTD binding in the vicinity. As for F1579, the diminution observed in Na_V_1.4 experiments contradicts our findings whereby no significant effect on the VTD concentration required for 10% delayed activation was observed for the F1760A mutation, although F1760Y caused a slight increase. For the peak currents, the diminished values caused by the L1462S, F1465Y, and F1760A mutations are consistent with the corresponding A/K mutations in Na_V_1.4 experiments (Supporting Table S1). Only the I1466T mutation was unaffected, compared to the I1284A mutation at Na_V_1.4, which is understandable considering the relatively conservative nature of the isoleucine to threonine mutation. Overall, the Na_V_1.4 and Na_V_1.5 results were consistent with the exception of the effect of the F1760A mutation.

### 2.14. Limitations of the Method

Although our calculations were successful in identifying the putative binding site of VTD on Na_V_1.5, and experimental shortcomings may have affected the identification of the exact binding configuration of VTD at this site and the corresponding ΔΔG values calculated. The resolution of the starting structure was 3.3 Å, which may have resulted in non-ideal conformations for some of the side chains found to be important in this study. The structure was refined with cryo-EM densities to ameliorate this potential issue. In addition, some interactions, including π-cation and π–π stacking, are not explicitly represented in Rosetta. As a result, the poses whereby the Ring 1 charged nitrogen is in the vicinity of aromatic residues, such as Y1767 or F1760, may have non-optimal geometries or diminished energy contributions coming from these interactions.

Other potential issues with the scoring are the effects of solvation or hydrogen bonding. VTD bears six -OH groups that can form hydrogen bonds with water molecules or the pore residues of the protein depending on its binding configuration. However, the pore-lining residues of Na_V_1.5 are mostly hydrophobic, which may suggest that these -OH groups can be paired with water molecules in the pore instead of protein residues. Since the Rosetta score function can model the effect of solvation only implicitly, the ΔΔG values may not accurately represent the solvation or the hydrogen bonding terms between VTD and the Na_V_1.5 residues.

In addition to the scoring-related factors, the ΔΔG calculations were run without relax calculations to prevent large-scale rearrangement of the protein side chain and VTD residues, which may have resulted in slight variations in the backbone configurations that might have been caused upon VTD binding to the unbound state of the protein.

Finally, despite the high-throughput nature of automated patch clamping, we were only able to obtain a few qualifying cells for some mutations. This reflects our stringent quality control criteria and results in slightly higher measurement error for some of the variants’ measured VTD binding properties.

## 3. Conclusions

Our results suggest that VTD binding at a solvent-exposed mouth site closer to the opening of the ion pore at the cytoplasmic face lined by the residues L409 and I1466 at the bottom is more favorable than a binding site deeper in the pore. This finding is supported by the lack of a significant change caused by mutations deeper in the pore site, and the effect of mouth site mutations that could have no binding effect if VTD bound to a site deeper in the pore. In the binding mode identified for the mouth site, the DMP group forms mostly hydrophobic interactions with the residues that form the bottom of the binding pocket, and Ring 1, with its charged nitrogen group, is exposed to the solvent. This binding conformation would also allow for hydrogen bonding between the VTD -OH groups and water, which otherwise would be buried in a highly hydrophobic pore with no interaction partners. In conclusion, our calculations identified a binding site hitherto not identified for the binding of VTD on Na_V_1.5 supported by electrophysiology measurements. Our findings do not exclude the possibility of VTD binding at other sites of Na_V_1.5, but only present an alternative binding site that can be relevant to delayed activation induced by VTD. The existence of an alternative binding site is consistent with the dual mode of action shown by this molecule that allows VTD to block single channel current and induce delayed activation. Knowledge of this alternative binding site potentially allows manipulation of one mode of action without necessarily altering the other at the same time. These results will inform the design of VTD analogs that can be used for therapeutic purposes and help us better understand the allosteric interactions between VTD and other sites that affect Na_V_1.5 activity.

## 4. Methods and Materials

### 4.1. Structure Preparation and Refinement

The human cardiac sodium channel structure with a bound quinidine molecule (PDB ID: 6LQA) was used for the calculations [19]. The ligand and the glycan molecules were removed from the structure for the refinement steps. The structure was relaxed using Rosetta *FastRelax* with cryo-EM restraints [20,21] using the electron density map deposited to PDB as a restraint with the *ref2015* score function [22]. A *dens_wt* (density weight) of 35 was used for the refinement calculations, followed by B-factor fitting with 50 iterations. A total of 100 relaxed structures were generated. The quality of the lowest-scoring structure selected by total score was inspected through the MolProbity server [23], whereby an improvement of the Ramachandran-favored residues (91.35% to 95.72%) and Z-scores was observed (−4.19 to 0.14). This refined structure was selected as the Na_V_1.5 protein model for the docking calculations.

### 4.2. Ligand Docking to the Mouth and Pore Sites of Na_V_1.5

BCL was used to create a conformer library of VTD that resulted in a total of 100 conformers [24]. These conformers were used for the following docking calculations. The *molfile_to_params.py* script embedded in Rosetta was used to generate the .params files necessary for the docking calculations with a total charge of +1 on the molecule. The center of the grid box was defined as the approximate middle point of E417 atom OE1 and N406 atom OD1 for the mouth site and the approximate middle point of F1760 and F1418 CZ atoms for the pore site. The *ligand.wts* score function weights were used for the docking calculations [25,26]. The box size was set to 20 Å, and the grid size was set to 45 Å to accommodate the large size of the VTD molecule and to cover a broad area at either docking site. A total of 10,000 poses were generated for each docking site and the interface scores were considered for the ranking and analysis of the poses.

### 4.3. Clustering and Analysis Based on the Interface Scores

The ligand RMSD values were calculated as the 3D distance between the lowest-scoring pose and all the other poses using aligned protein structures. The interface scores were used to plot the score versus RMSD plots. Ligand clustering was performed with the k-means method using an in-house script, and a total of five clusters were chosen for each calculation. All plots were generated with the *matplotlib.pyplot* module of Python. The five selected clusters for each site were inspected visually to identify the key interactions between the VTD poses and the Na_V_1.5 residues.

### 4.4. VTD ΔΔG Calculations with WT and the Mutant Na_V_1.5

The Rosetta *ddGScan* application was used to screen for the mutants that showed the best correlation with the experimental binding data. Repacking and relax for both the bound and unbound states were switched off to reduce the time necessary for the calculations, and to prevent perturbations at the binding site that may affect the results. Specifically, the Rosetta scores were calculated in the absence and presence of the VTD molecule (ΔG) prior to and after the introduction of a particular mutation. The ΔG_mutant_ − ΔG_WT_ difference was used as the ΔΔG value for each mutation.

Correlation analysis was performed with in-house Python scripts using the numpy module of Python. The R^2^ values were calculated using the ΔΔG values and the measured VTD 10% delayed activation concentration values. The ten structures that presented the best correlation scores were inspected visually to identify the similarities among the poses. The highest-correlation pose was used for all the visual analyses.

### 4.5. Mutagenesis and Stable Cell Line Generation

*SCN5A* mutagenesis and Human Embryonic Kidney (HEK) 293 stable cell line generation were performed, as previously described [16]. Briefly, mutations were introduced with a QuikChange Multi Lightning kit (Agilent) in a small “zone” plasmid, sequence verified, and transferred with restriction digestion to a plasmid containing full length *SCN5A*:IRES:mCherry. This plasmid was integrated into HEK293 “landing pad” negative selection cells [27], the landing pad expression was induced with 1 µg/mL doxycycline, and stable cells were generated by selection with 10 nM AP1903 and 100 µg/mL blasticidin for 7–10 days. Cell lines were screened by flow cytometry to ensure a high percentage of mCherry+, BFP− cells, which indicated successful plasmid integration and expression.

### 4.6. Veratridine Binding Measurements

The cells were assayed on the SyncroPatch 384 PE, as previously described [16], with the exception of VTD addition. Following the measurement of baseline parameters, including peak current density, the cells were exposed to a repetitive 200 ms depolarizing pulse from -120 mV to −30 mV, repeating every 5 s. Following the baseline measurements, increasing doses of VTD were applied (0, 0.5, 1.58, 5, 15.8, and 50 µM), followed by a full channel blocker (150 µM tetracaine) with a 4 min gap between each addition. Cells with a seal resistance >500 mOhm, a capacitance between 5 and 30 pA, and a peak current greater than 500 pA were included in the analysis. The mean late current at 200 ms at each time point was measured and normalized to peak current. The normalized late current values for each variant were averaged across replicate cells and linear interpolation between the log10(VTD concentration), and the normalized late current was used to estimate the concentration necessary to cause 10% late current. For mutants that severely disrupted VTD binding, the highest applied VTD dose was not sufficient to cause 10% late current, and therefore this estimation could not be performed. For these mutants, the dose to cause 10% late current was reported as >50 µM.

## Figures and Tables

**Table 1:** The scores of the representative poses of the five clusters at the mouth site and the pore site calculated based on 10,000 docking poses. The pose numbers represent the corresponding pose at each site. All scores are reported in Rosetta Energy Units (REUs).

Pose Number	Mouth Site Score	Pore Site Score
Pose 1	−15.4	−13.9
Pose 2	−13.4	−15.4
Pose 3	−14.6	−16.6
Pose 4	−14.5	−16.0
Pose 5	−15.9	−15.9

**Table 2:** The effect of eleven mutations on the measured peak densities, number of cells used for the measurements, and the concentration of VTD required to induce a 10% late current (% of peak current). The standard errors of the mean could not be calculated for the measurements or for the measurements where the necessary concentration was larger than 50 µM.

Mutation	Peak Density (Norm. to WT)	Number of Cells	[Veratridine] for 10% Late Current (µM) ± S.E.
WT	100 ± 6.4	15	8.2 ± 0.4
E417A	111.5 ± 14.6	7	7.8 ± 0.6
E417R	94 ± 9.6	9	31.2 ± 1.9
L409S	61.3 ±16	2	>50
F942A	48.2 ± 5.1	6	10.8 ± 0.9
F942Y	88.5 ± 8	7	11.0 ± 0.8
L1462S	4.2 ± 0.7	1	6.9
F1465Y	50 ± 5.7	5	24.5 ± 2.1
I1466T	117.7 ± 12.8	5	>50
F1760A	59.4 ± 8	6	7.6 ± 0.5
F1760Y	15.2 ± 1.6	1	18.6
I1771T	54.6 ± 7.3	8	9.8 ± 0.8

## Data Availability

All data is available in the manuscript and the Supporting Information Files.

## Supplementary Materials

The supporting information can be downloaded at: https://www.mdpi.com/article/10.3390/ijms23042225/s1.
