# Supplementary material for: Veratridine Can Bind to a Site at the Mouth of the Channel Pore at Human Cardiac Sodium Channel NaV1.5
Source: Int J Mol Sci. 2022 Feb 17;23(4):2225. doi: 10.3390/ijms23042225 (PMC8878851; doi:10.3390/ijms23042225)
Supplement: Supplementary file 1 [file ijms-23-02225-s001.zip › ijms-1560702-supplementary.pdf]

International Journal of
Molecular Sciences

MDPI

Supporting Information

Supporting Tables

Supporting Table S1: **Baseline Electrophysiological Parameters.** Three parameters are shown: peak current density percentage (normalized so that wildtype is 100%), the voltage of 1/2 activation, and late current (presented as a percentage of peak current). For each parameter, mean, standard error, and the number of included cells are presented. Due to quality control filtering criteria (*i.e.* cells losing a high-quality seal over the course of the measurements), the number of cells for each parameter varies.

	Peak Density		$V_{1/2}$ Activation		Late Current (% of Peak)	
**Mutation**	**Mean (SE)**	**# Cells**	**Mean (SE)**	**# Cells**	**Mean (SE)**	**# Cells**
WT	100 (6.4)	87	-47.9 (0.9)	43	2.2 (0.5)	15
E417A	111.5 (14.6)	22	-45.1 (3.6)	4	1.1 (0.4)	7
E417R	94 (9.6)	24	-52.4 (2.2)	7	1.9 (0.4)	9
L409S	61.3 (16)	11	-40.6 (1.4)	8	2.1 (0.9)	2
F942A	48.2 (5.1)	32	-41.1 (1.1)	19	2.7 (0.8)	6
F942Y	88.5 (8)	31	-49.8 (1.6)	10	2.9 (1)	7
L1462S	4.2 (0.7)	27	-19.9 (0.8)	22	6.9 (*)	1
F1465Y	50 (5.7)	34	-42.2 (1.3)	22	1 (0.2)	5
I1466T	117.7 (12.8)	23	-34.6 (0.6)	4	1.4 (0.3)	5
F1760A	59.4 (8)	40	-37.5 (0.8)	41	3.1 (1.5)	6
F1760Y	15.2 (1.6)	49	-43.8 (1.3)	28	4.1 (*)	1
I1771T	54.6 (7.3)	46	-32.8 (1)	31	3.6 (1.2)	8

Supporting Figures

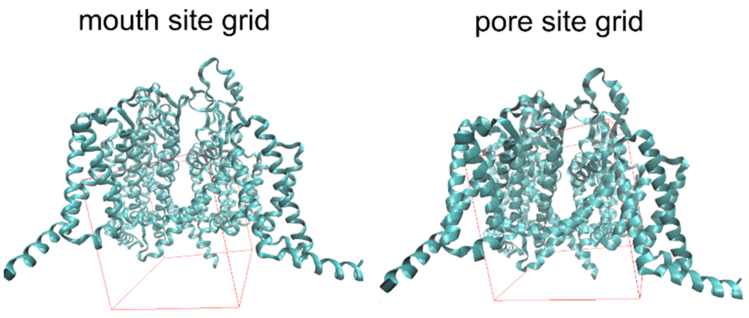

Supporting Figure S1: Visual representation of the mouth and pore site grids selected for the docking calculations.

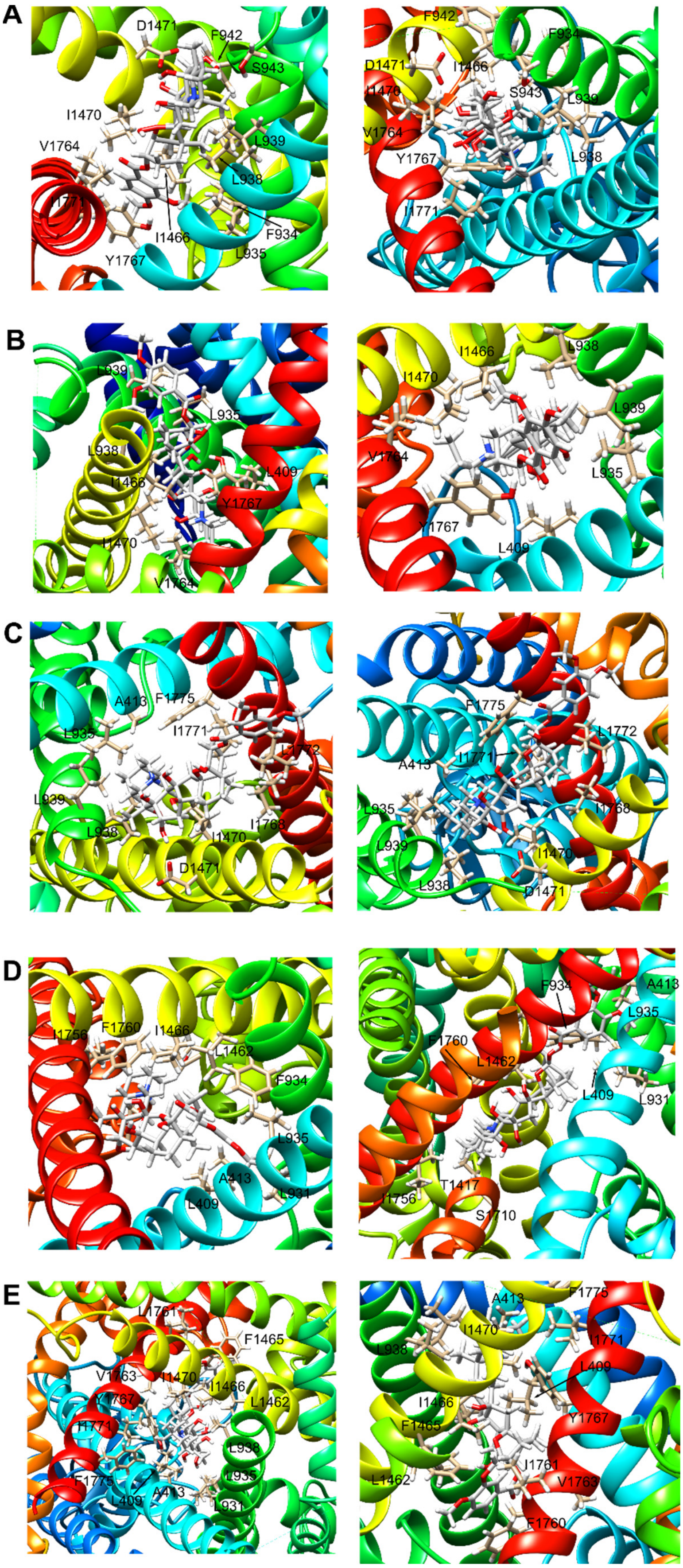

Supporting Figure S2: The mouth side poses viewed from different angles for Pose 1 (A), Pose 2 (B), Pose 3 (C), Pose 4 (D), and Pose 5 (E). Veratridine (VTD) is shown in gray.

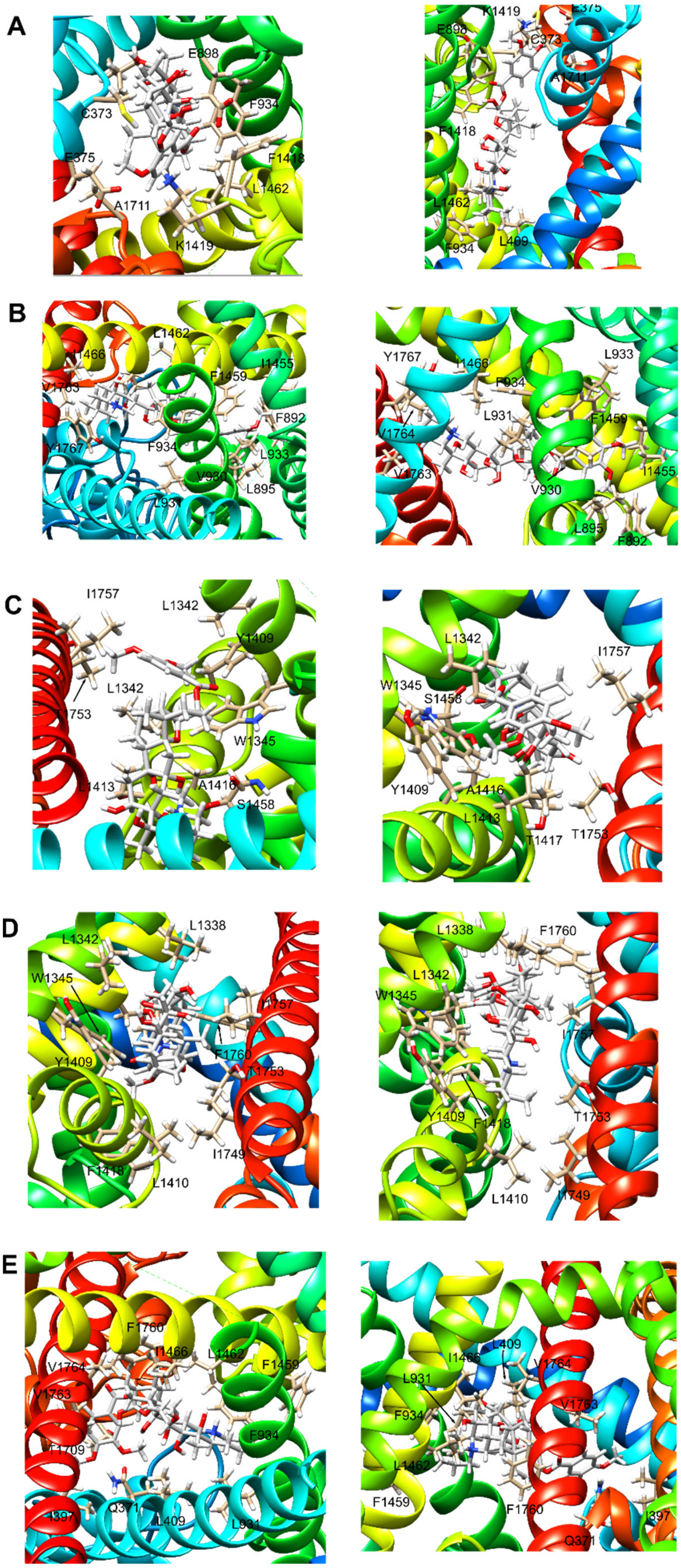

Supporting Figure S3: The pore site poses viewed from different angles for Pose 1 (A), Pose 2 (B), Pose 3 (C), Pose 4 (D), and Pose 5 (E). Veratridine (VTD) is shown in gray.

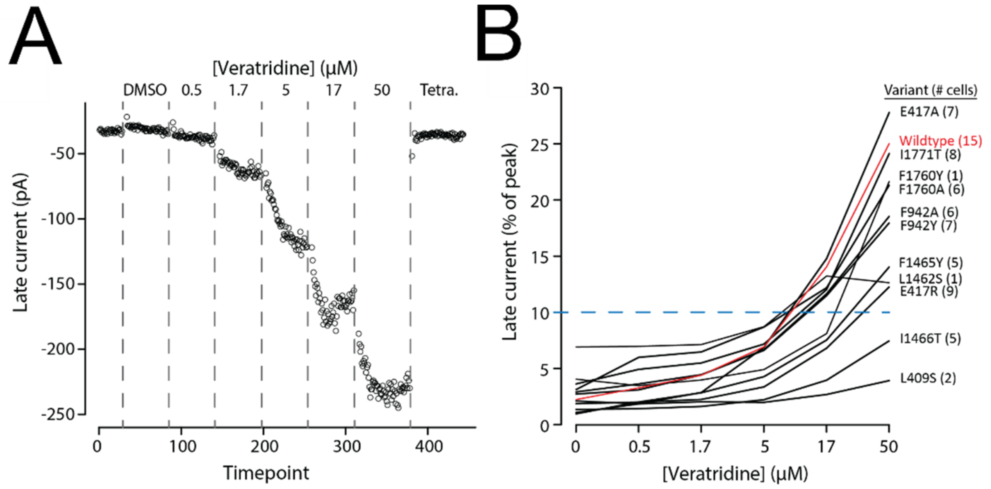

Supporting Figure S4: A) Example data of a wildtype cell treated with increasing concentrations of veratridine, followed by a high dose of the sodium channel blocker tetracaine. Raw late current is plotted in pA. Timepoints were taken every 5s. B) Late current (% of peak current) dose-response curves for response to veratridine at different Nav1.5 variants and the WT. The number of cells for each variant is listed in parentheses. These data were used to calculate the veratridine concentration required for 10% late current (percent of peak current) in Table 2.

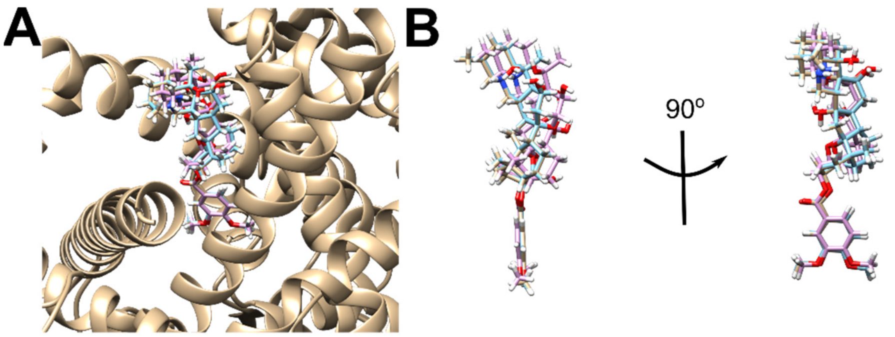

Supporting Figure S5: A) The three poses that had the best correlation factors at the mouth site based on the ΔΔG calculations shown bound at the mouth site, B) Overlap of the three ligand configurations shown from two different angles. Beige, blue, and pink correspond to the three different VTD configurations from the docking calculations whereby blue color indicates the highest-correlation pose, pink color indicates the second highest-correlation pose, and beige indicates third highest-correlation pose.
