# Supplementary figures and images for: Veratridine Can Bind to a Site at the Mouth of the Channel Pore at Human Cardiac Sodium Channel NaV1.5
Source: Int J Mol Sci. 2022 Feb 17;23(4):2225. doi: 10.3390/ijms23042225 (PMC8878851; doi:10.3390/ijms23042225)

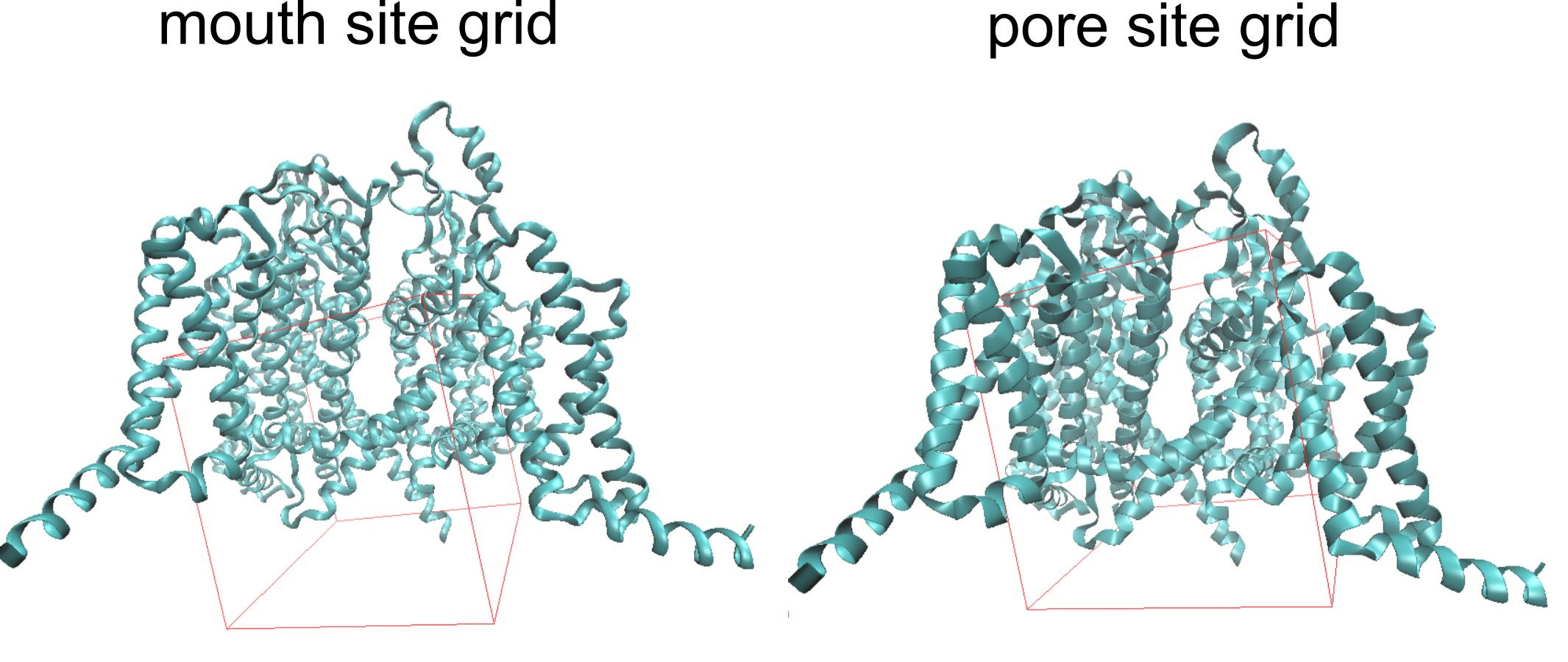

Supplement: Supplementary file 1 [file ijms-23-02225-s001.zip › FigS1.png]

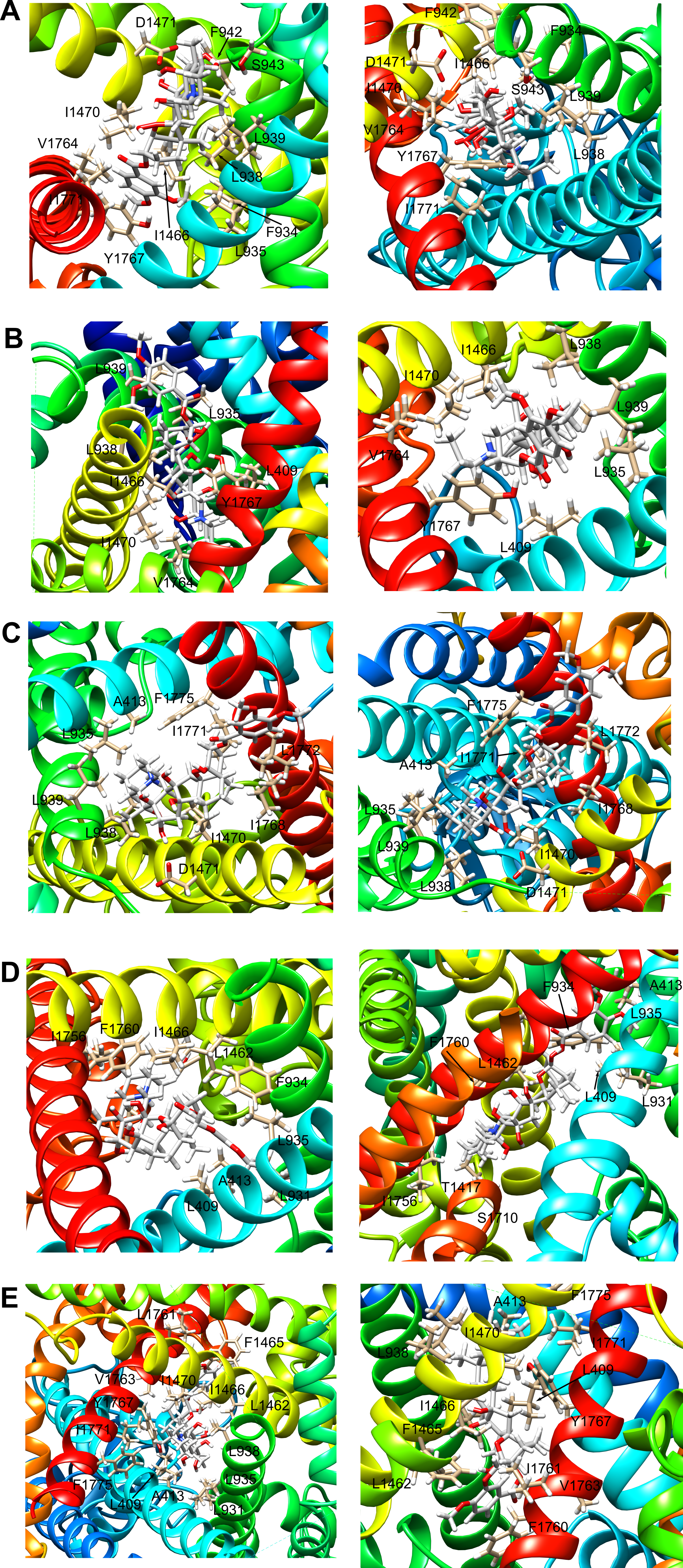

Supplement: Supplementary file 1 [file ijms-23-02225-s001.zip › FigS2.png]

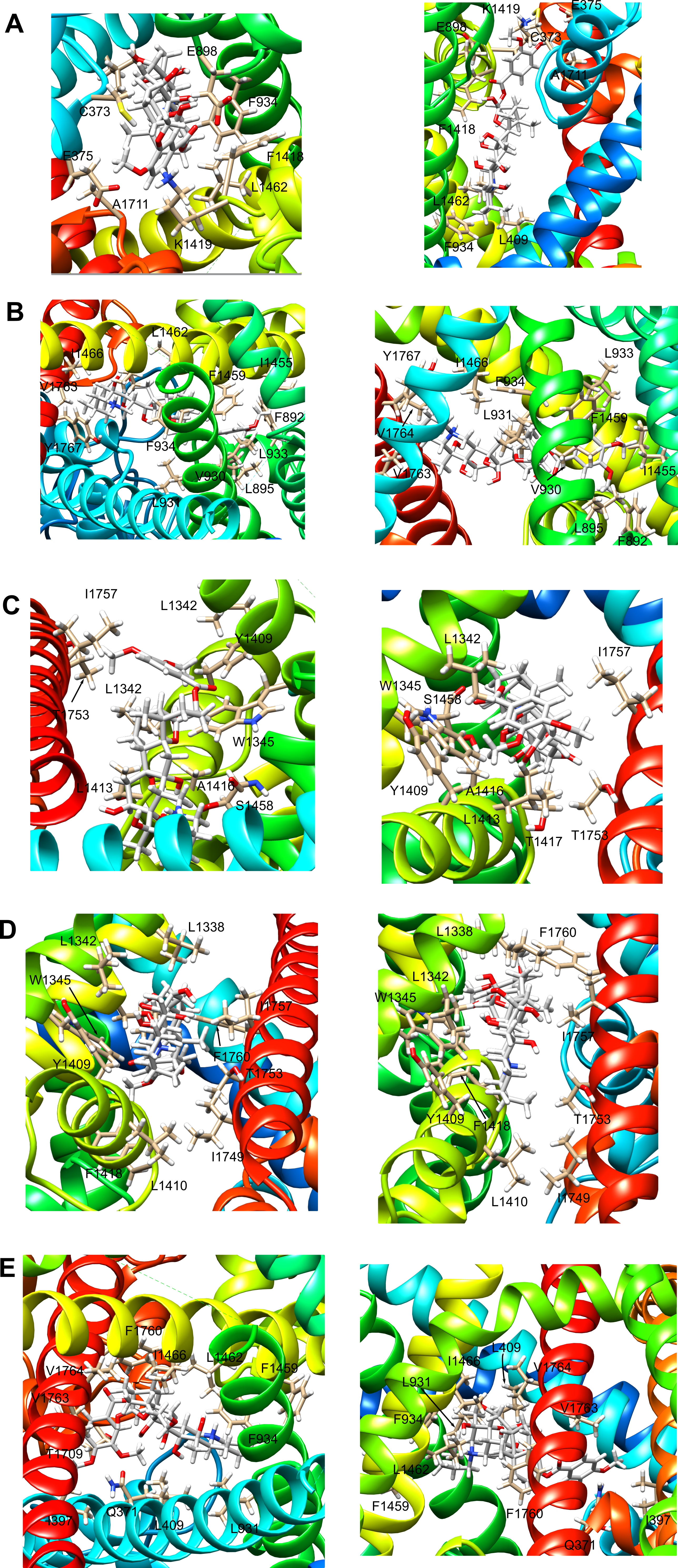

Supplement: Supplementary file 1 [file ijms-23-02225-s001.zip › FigS3.png]

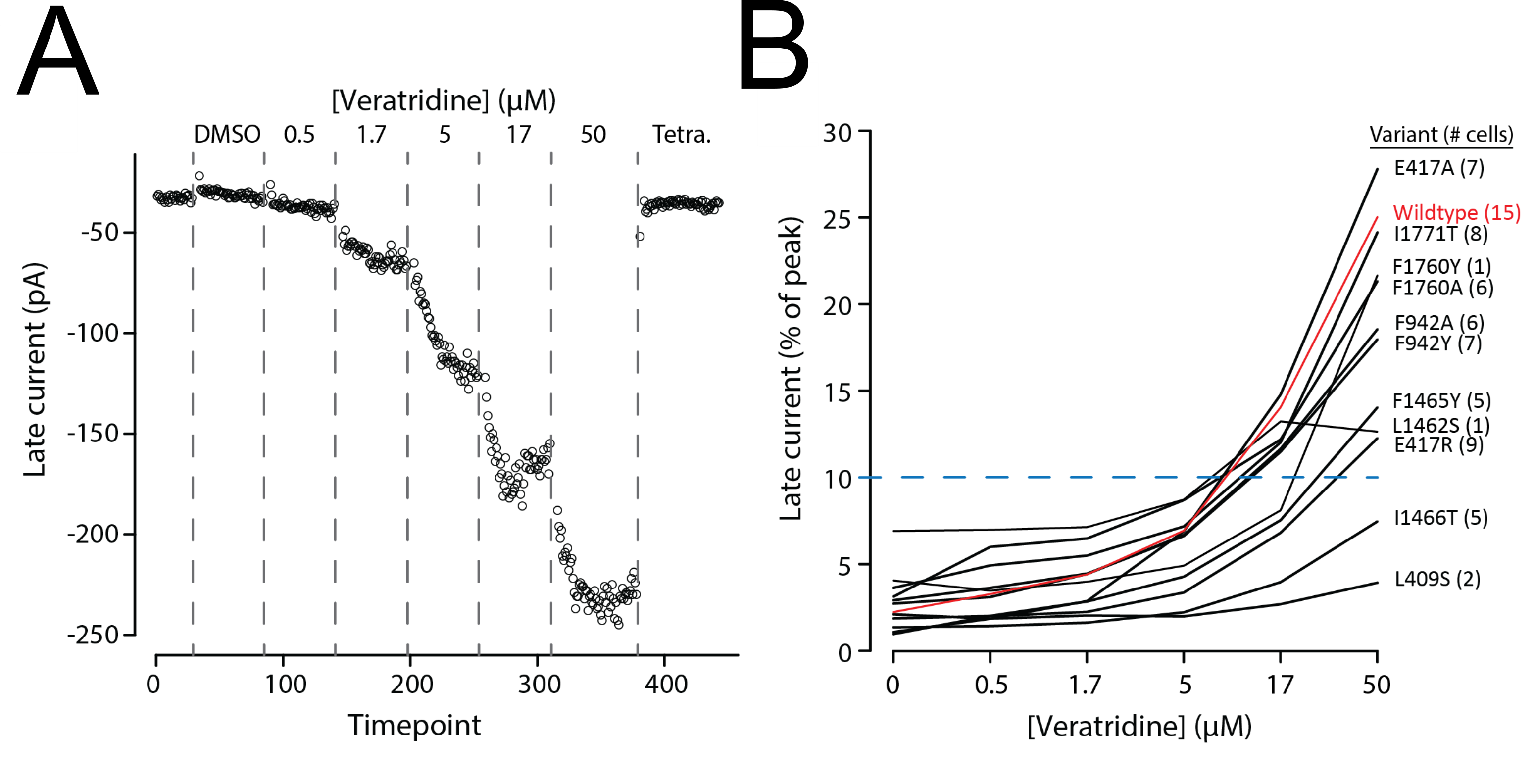

Supplement: Supplementary file 1 [file ijms-23-02225-s001.zip › FigS4.png]

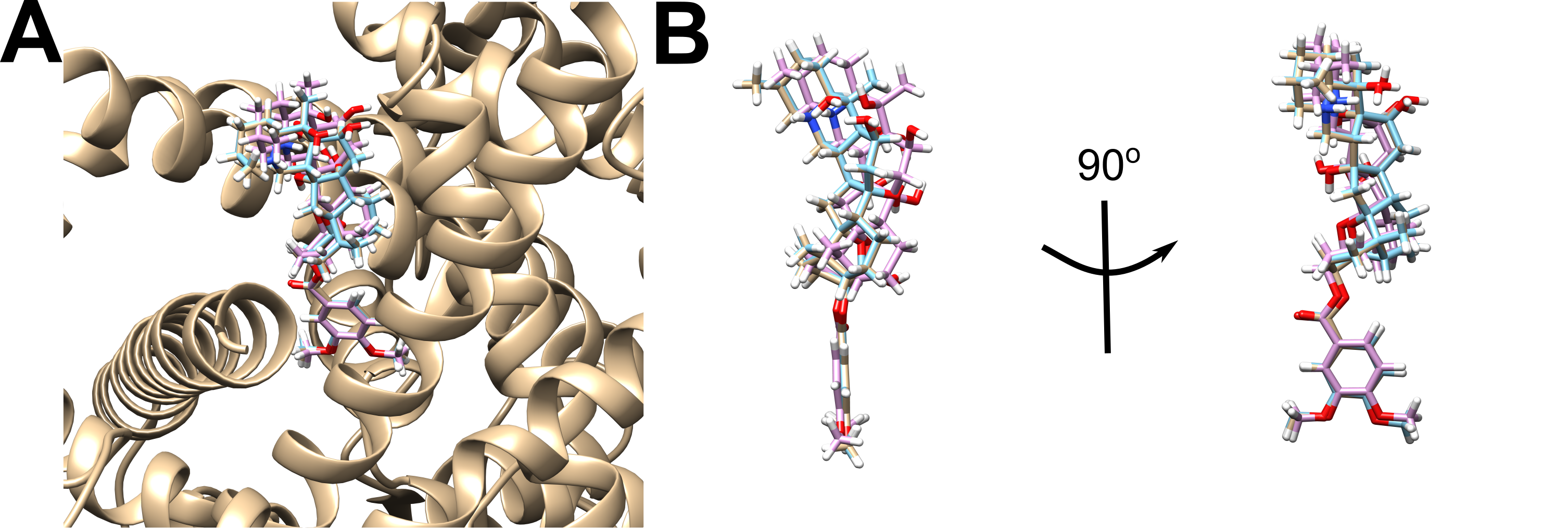

Supplement: Supplementary file 1 [file ijms-23-02225-s001.zip › FigS5.png]
